# Matrix metalloproteinases: Expression, regulation and role in the immunopathology of tuberculosis

**DOI:** 10.1111/cpr.12649

**Published:** 2019-06-14

**Authors:** Naveed Sabir, Tariq Hussain, Mazhar Hussain Mangi, Deming Zhao, Xiangmei Zhou

**Affiliations:** ^1^ Key Laboratory of Animal Epidemiology and Zoonosis, Ministry of Agriculture, National Animal Transmissible Spongiform Encephalopathy Laboratory, College of Veterinary Medicine China Agricultural University Beijing China

**Keywords:** Host‐directed therapy, Immunopathology, MMPs, *Mtb*, Signalling pathways

## Abstract

*Mycobacterium tuberculosis* (*Mtb*) leads to approximately 1.5 million human deaths every year. In pulmonary tuberculosis (TB), *Mtb* must drive host tissue destruction to cause pulmonary cavitation and dissemination in the tissues. Matrix metalloproteinases (MMPs) are endopeptidases capable of degrading all components of pulmonary extracellular matrix (ECM). It is well established that *Mtb* infection leads to upregulation of MMPs and also causes disturbance in the balance between MMPs and tissue inhibitors of metalloproteinases (TIMPs), thus altering the extracellular matrix deposition. In TB, secretion of MMPs is mainly regulated by NF‐κB, p38 and MAPK signalling pathways. In addition, recent studies have demonstrated the immunomodulatory roles of MMPs in *Mtb* pathogenesis. Researchers have proposed a new regimen of improved TB treatment by inhibition of MMP activity to hinder matrix destruction and to minimize the TB‐associated morbidity and mortality. The proposed regimen involves adjunctive use of MMP inhibitors such as doxycycline, marimastat and other related drugs along with front‐line anti‐TB drugs to reduce granuloma formation and bacterial load. These findings implicate the possible addition of economical and well‐tolerated MMP inhibitors to current multidrug regimens as an attractive mean to increase the drug potency. Here, we will summarize the recent advancements regarding expression of MMPs in TB, their immunomodulatory role, as well as their potential as therapeutic targets to control the deadly disease.

## 1 INTRODUCTION

Tuberculosis (TB) is a major threat to public health worldwide, and it continues to kill more than 1.5 million people annually.^1^ Statistically, developing countries account for 90% of the tuberculosis cases worldwide.^2^ *Mycobacterium tuberculosis* (*Mtb*) is a successful intracellular pathogen. Once infected with *Mtb*, host innate immune response is activated and macrophages constitute an important part of the innate immune system.^3^ Importantly, they play a critical role in recognizing, responding and reacting to *Mtb* infection.^4^ Phagocytosis of *Mtb* by macrophages can be triggered by non‐specific pinocytosis or by the activation of specific receptors. Moreover, *Mtb* can also be recognized through pattern recognition receptors (PRRs) such as Toll‐like receptors (TLRs) and Nod‐like receptors (NLRs).^5^ This interaction of *Mtb* and macrophages eventually activates inflammatory response.^6^

Lungs are the primary site of *Mtb* infection, and pulmonary TB is characterized by granulomatous inflammation and destruction of lung parenchyma. The host immune response limits the spread of *Mtb* and walls off the bacteria in dense cellular masses known as granulomas or tubercular lesions.^7^, ^8^ Although host innate immune response is one of the important determinants of the disease, the possible outcome of the infection varies among susceptible individuals and the factors involved therein are not well understood.^9^, ^10^ Recent studies have suggested a new concept of TB immunopathology that directly involves inhibition of matrix metalloproteinase (MMP) activity to hinder matrix destruction and reduce the morbidity and mortality associated with TB.^11^, ^12^

Matrix metalloproteinases (MMPs, also known as matrixins) are secreted or membrane‐bound endopeptidases belonging to the metzincin superfamily, collectively capable of degrading all components of ECM. The prefix “metallo‐” refers to the reliance of these enzymes on zinc ions to carry out the hydrolysis of protein substrates, and their structure has been reviewed in detail.^13^, ^14^ The first MMP was reported by Gross and Lapiere in 1962 as a collagenase engaged in tail resorption during the tadpole metamorphosis.^15^ Currently, MMPs consist of 23 members in human and are expressed in almost all organs and tissues.^16^ These enzymes have key roles in inflammatory cell migration, tissue repair, chemokine and cytokine signalling, degradation of matrix and non‐matrix proteins, pathogenesis of various diseases and modulation of immune responses.^17^, ^18^, ^19^, ^20^, ^21^ MMPs can be broadly classified on the basis of substrate specificity into collagenases (MMP‐1, MMP‐8 and MMP‐13), gelatinases (MMP‐2 and MMP‐9), stromelysins (MMP‐3, MMP‐10 and MMP‐11), elastases (MMP‐7 and MMP‐12) and membrane‐type MMPs (MT‐MMPs; MMP‐14, MMP‐15, MMP‐16 and MMP‐17) which are surface anchored.^22^

Most of the MMPs are secreted as inactive zymogens called proMMPs which have a cysteine switch motif coordinating with Zn^2+^ in catalytic domain.^23^ In vitro, these proMMPs can be activated by chemical agents, such as sodium dodecyl sulphate, oxidized glutathione and thiol‐modifying agents^24^; however, in vivo activation of proMMPs is more complicated and is conducted by other MMPs or other classes of proteinases such as plasmin and neutrophil elastases.^25^ In healthy tissues, MMPs are occasionally expressed and their biological activity is tightly regulated by various mechanisms. Activity of activated MMPs is regulated by endogenous inhibitors called tissue inhibitors of metalloproteinases (TIMPs) that bind active and latent forms of MMPs.^26^

Matrix metalloproteinases activity is implicated in non‐infectious and chronic lung diseases such as asthma and COPD.^27^, ^28^, ^29^ *Mtb* infection leads to disturbance in the balance between MMPs and TIMPs, and also alters extracellular matrix deposition as well as the cell behaviour of monocyte‐microglial networks.^30^, ^31^ MMPs are secreted by *Mtb*‐infected macrophages and monocytes, and also by uninfected stromal cells stimulated through intercellular networks.^32^ Many studies have demonstrated the involvement of MMP‐1, the major human collagenase, and its activator MMP‐3 in driving pathology in pulmonary TB.^30^, ^33^, ^34^ In this review, we will focus on the recent studies demonstrating the immunomodulatory roles of MMPs and their potential as therapeutic targets to hamper the pulmonary matrix destruction and reduce the morbidity and mortality associated with TB.

## 2 EXPRESSION OF MMPS IN TB

The majority of MMPs are expressed in diseased conditions wherein the tissues are inflamed and undergo repair and remodelling, while some of the members such as MMP‐2, MMP‐19 and MMP‐28 are evident in normal tissues indicating their roles in homeostasis.^35^ Many immune cells express low levels of MMPs in the resting state, and expression of MMPs is upregulated by exogenous stimuli, cytokines and cell‐cell interaction.^36^ This regulation is mainly carried out by TIMPs as unstimulated human peripheral blood monocytes, B cells and T cells express higher levels of TIMP‐1, TIMP‐2 and TIMP‐4.^37^ Pulmonary epithelial cells are also a significant source of MMPs as they express many MMPs including MMP‐1, MMP‐2, MMP‐7 and MMP‐9.^38^ In many pathological conditions, cell migration is closely linked to degradation of the ECM and the activated MMPs are considered as a prerequisite for invasion and metastasis of cancerous cells.^39^

Many studies have analysed the expression of MMPs in the pathophysiology of TB (Table 1). Infection of THP‐1 cells with *Mtb* leads to increased expression of MMP‐9. This MMP‐9 induction is regulated by receptor‐mediated signalling pathways.^40^ In TB patients, plasma concentrations of various MMPs may vary between the genders and this expression may not associate with the severity of the disease. Sathyamoorthy et al found significantly higher plasma concentrations of MMP‐1 and MMP‐8 in male TB patients as compared to females. This increased concentration of the MMPs was inversely correlated with body mass index.^41^ Similarly, plasma MMP‐3 was also significantly higher in men as compared to women in a number of clinical conditions including both infectious and non‐infectious diseases.^42^ MMPs, like MMP‐1, cause lung extracellular matrix destruction, and MMP‐10 is known as a key activator of MMP‐1. In a recent study, MMP‐10 secretion was increased in *Mtb*‐infected macrophages while inhibition of MMP‐10 activity decreased collagen breakdown. MMP‐10 expression was also increased in both induced sputum and bronchoalveolar lavage fluid (BALF) as compared to control subjects and patients with other respiratory diseases.^43^ This *Mtb*‐driven MMP‐10 secretion was inhibited in a dose‐dependent manner by p38 and extracellular signal–related kinase mitogen‐activated protein kinase blockade. In vivo and in vitro, *Mtb* infection leads to increased expression and activity of MMP‐1, MMP‐2, MMP‐3 and MMP‐9.^44^ This study also reported the involvement of miR‐223 in MMP expression through BMAL1 modulation. Azikin et al evaluated the levels of MMP‐9 in children who lived in the same house with a person having active TB.^45^ There were no significant differences between the expression levels of MMP‐9 in the group of exposed and *Mtb* infected children, and the levels of MMP‐9 were not influenced by sex, age, nutritional status and the status of BCG immunization. In a related study, *M avium* also induced the secretion of MMP‐1 in duodenal biopsy tissues, as well as in blood samples as compared to negative controls.^46^ This induction of MMP‐1 by *M avium* in duodenal tissue suggests that mycobacteria might contribute to the epithelial disruption commonly seen in enteropathies. Systemic levels of various MMPs may reflect the severity of disease in TB patients. Kumar et al reported elevated levels of circulating MMP‐1, MMP‐2, MMP‐3, MMP‐7, MMP‐10 and MMP‐12 in TB patients having diabetes mellitus as compared to patients having TB only.^47^ Moreover, anti‐tuberculosis therapy with metformin was associated with a significant reduction in the levels of MMP expression. Gao et al compared the effect of minimally invasive operation and open surgery on the serum IL‐1β, MMP‐1 and MMP‐13 in patients with senile spinal tuberculosis.^48^ The serum values of IL‐1β, MMP‐1 and MMP‐13 after surgery were lower than those of before treatment.

**Table 1:** Expression of MMPs and TIMPs in TB

Species examined	Type of tissue/cells examined	Upregulation of MMPs/TIMPs	References
Human	THP‐1 cells	MMP‐9	40
Human	Monocytes	MMP‐1 and MMP‐3	141
Human	Blood	MMP‐1 and MMP‐8	41
Human	Plasma	MMP‐3	42
Human	Co‐culture model of the blood‐brain barrier	MMP‐9	43
Human	Serum	MMP‐1 and MMP‐13	48
Mouse	Blood and macrophages	MMP‐1, MMP‐2, MMP‐3 and MMP‐9	44
Human	Lung tissue model	MMP‐1, MMP‐3, MMP‐9 and MMP‐12	12
Human	Blood	MMP‐9	45
Human	Duodenal biopsy tissues and blood	MMP‐1	46
Human	Blood	MMP‐1, MMP‐2, MMP‐3, MMP‐7, MMP‐10 and MMP‐12	47
Human	Cerebrospinal spinal fluid	MMP‐1 and MMP‐3	30, 51
Human and mouse	Lung tissues	MMP‐8	165
Human	Lung biopsies and macrophages	MMP‐8	101
Human	Brain biopsies	MMP‐9	52
Mouse	Brain biopsies	MMP‐9	53
Human	Bronchial epithelial cells	MMP‐9	80
Human	Sputum and bronchoalveolar lavage fluid (BALF)	MMP‐1	34
Human	Pleural fluid	MMP‐2 and MMP‐9 TIMP‐1	62
Human	Plasma and THP‐1 cells	TIMP‐1	65
Human	Macrophages	MMP‐1 and MMP‐7	66
Human	Monocyte‐derived Macrophages	MMP‐1 and MMP‐3	102
Human	Sputum and macrophages	MMP‐14	140
Human	Blood	MMP‐1, MMP‐2, MMP‐3, MMP‐8 and MMP‐9 and TIMP‐1, TIMP‐12	63

Like other tissues of the body, inflammation of central nervous system (CNS) also results in increased MMP secretion and it can also affect the permeability of blood brain barrier (BBB).^49^, ^50^ Increased expression of MMP‐1 and MMP‐3 has been reported in the patients with TB of CNS.^30^, ^51^ Similarly, in a recent study, MMP‐9 upregulation has been noticed in the brain biopsies of the patients having TB meningitis.^52^ This enhanced activity of MMP‐9 in the brain tissues may be involved in the damage of BBB, oedema and the inflammatory cell exudation. Li et al analysed the expression of MMP‐9 in the pathophysiological process of TB meningitis in a mouse model.^53^ The data exhibited elevated expression and activity of MMP‐9 as compared to control group. Although many studies have reported the differential expression of MMPs in TB, there is still a need for comprehensive MMP expression profiling in different immune cell lineages under resting and activated states, and in response to *Mtb* and other bacterial challenges.

## 3 REGULATION OF MMP EXPRESSION AND ACTIVITY

Since MMPs might cause significant damage to the host tissues, therefore, their expression is strictly regulated. This regulation involves several levels including gene expression, zymogen activation, compartmentalization and inhibition of active enzyme.^35^ Initially, MMPs are believed to be regulated at the transcriptional level by a variety of physiological factors including cytokines, tumour promoters, growth factors, hormones, chemokines and cell‐cell or cell‐ECM communications.^54^ MMP promoters contain cis‐acting elements that can be bound and regulated by several transcription factors such as activator protein 1 and NF‐kB.^55^ Expression of MMPs is further regulated at the post‐transcriptional level.^56^ Studies also have uncovered the contribution of epigenetic modifications in regulation of MMPs.^57^ MMP activity is also controlled through their compartmentalization in a certain intracellular or extracellular location.^58^ In addition, many intracellular pathways have been discovered which are actively involved in MMP regulation.

### 3.1 Role of TIMPs in MMP regulation in TB

Tissue inhibitors of metalloproteinases are well known to downregulate the activity of MMPs by binding their latent and active forms. TIMPs are constitutively expressed in many tissue fluids including cerebrospinal fluid (CSF).^59^, ^60^ There is a considerable connection in the biochemical properties of various TIMPs, although there are some MMPs having specificity to substrates.^16^ Expression of MMPs and TIMPs in TB has been reported in many studies. Residual pleural thickening (RPT) is the most commonly seen complication related to pleural TB, and it may happen even after successful anti‐tuberculosis medication. TIMP‐1 is an endogenous inhibitor of MMPs and regulates MMP activity by forming 1:1 complexes with MMPs.^61^ In a previous study, higher levels of TIMP‐1 in pleural fluid were found to be responsible for the development of RPT while expression of MMP‐2 and MMP‐9 had no significant correlation to RPT.^62^ This indicates the role of TIMP‐1 in RPT, and its expression level may predict the occurrence of RPT in pleural TB. Other studies have also unveiled that levels of MMP‐1, MMP‐2, MMP‐3, MMP‐8 and MMP‐9, as well as TIMP‐1 and TIMP‐12, were significantly higher in TB patients as compared with healthy controls.^34^, ^63^, ^64^ Recently, expression level of TIMP‐1 in plasma has been reported as a potential biomarker for the diagnosis of TB. Moreover, Bacillus Calmette‐Guérin (BCG) and *M bovis* infection of THP‐1 cells also significantly enhanced the TIMP‐1 mRNA expression in a time‐dependent manner.^65^ In contrast, Rand et al reported increased secretion of MMP‐1 and MMP‐7, and decreased expression of TIMP‐1 in primary human macrophages infected with *Mtb.* 66 Downregulation of TIMP‐1 could lead to increased activity of MMP‐1 and MMP‐7 and more tissue destruction which can be seen as an *Mtb* strategy to replicate and spread to other tissues. It is obvious that the balance between MMPs and TIMPs regulates matrix turnover, wherein either a surplus of MMPs or a scarcity of TIMPs may cause excessive ECM degradation and tissue damage.

### 3.2 Kallikrein‐kinin system in MMP regulation

Kallikrein‐related peptidases (KLKs) are a subgroup of serine proteases with either trypsin‐like or chymotrypsin‐like activity. Tissue KLKs are expressed in various tissues and consist of 15 proteases, KLK1 to KLK15. KLKs perform many physiological and pathological functions. The kallikrein‐kinin system appears to play a direct role in promoting anti‐fibrotic responses and collagen degradation.^67^, ^68^ As KLKs are able to convert kininogens into bradykinin, these kinins then bind to bradykinin receptor 1 (B1R) and bradykinin receptor 2 (B2R). B1R is generally latent, but it is upregulated in inflammation or by the members of cytokine family including IL‐1β and TNF‐α, while B2R is constitutively expressed in many tissues of the body.^69^ Bradykinins play a pivotal role in the modulation of airway inflammation by stimulation of cytokine expression and recruitment of inflammatory cells.^70^, ^71^

**Figure 1:**
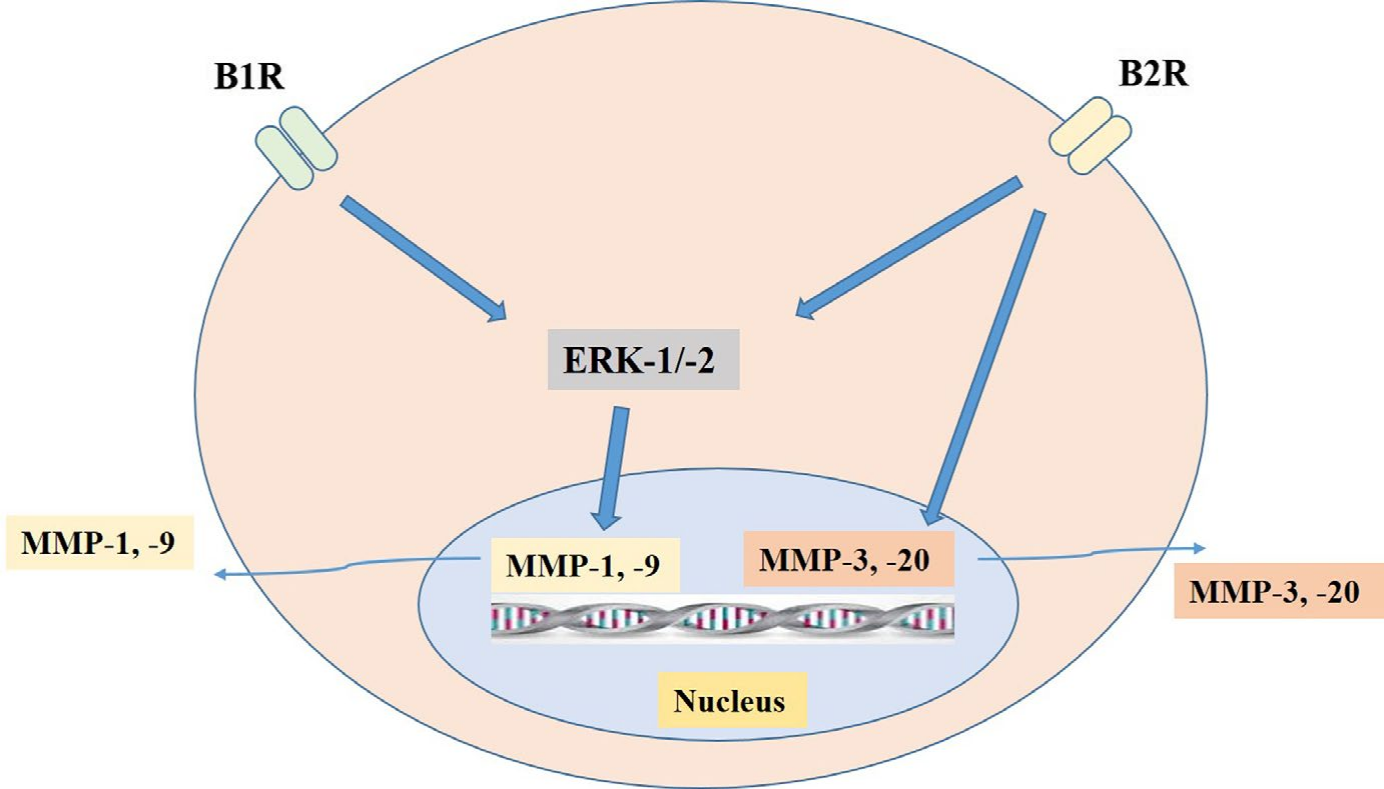
Role of bradykinin signalling in expression and regulation of MMPs in non‐TB conditions. Bradykinin stimulation can lead to activation of intracellular signalling pathways which in turn induces various MMP secretion. Both B1R and B2R can activate ERK1/2 kinases eventually leading to increased expression of MMP‐1 and MMP‐9 in breast cancer and tracheal smooth muscles, respectively. Similarly, exogenous bradykinin treatment of granulosa cells induced MMP‐3 and MMP‐20 explaining their role in ovulation

Many studies have investigated whether bradykinin stimulation induces release of MMPs in different tissues (Figure 1). For example, bradykinin treatment of isolated granulosa cells induced MMP‐3 and MMP‐20 expression explaining the role of bradykinin in ovulation in pigs.^72^ Pharmacological blockade or knockdown of B2R receptor (*B2^−/–^*) in mice and rats resulted in increased interstitial fibrosis, whereas transgenic mice expressing increased endogenous B2R showed reduced interstitial fibrosis. The increased interstitial fibrosis in *B2^–/–^* mice was associated with decreased activity of MMP‐2 suggesting the protective role of bradykinin and MMP‐2.^73^ Similarly, involvement of B2R in the release of MMP‐2 from tracheal smooth muscle cells of guinea pig has been reported.^74^ In a recent study, B1R agonist, Lys‐des[Arg9]‐bradykinin (LDBK), increased the proliferation of oestrogen‐sensitive breast cancer cells. B1R was also involved in the expression of MMP‐2 and MMP‐9 via ERK‐dependent pathway.^75^ Similarly, B2R has been reported to regulate MMP‐9 secretion via MAP kinases (ERK1/2) signalling in trabecular meshwork cells.^76^ On the other hand, MMPs can also regulate the function of KLKs.^77^ These studies suggest a potential interaction of KLKs and MMPs in various physiological and pathological conditions. It is likely that this B1R and B2R signalling is involved in the MMP secretion in TB but no study has been reported yet. Investigations of these signalling pathways may reveal bradykinin receptors as therapeutic targets in TB treatment.

### 3.3 IL‐12 and IL‐17 cytokines mediated induction of MMPs

Cytokines are the important regulators of immunity and inflammation. These cytokines including TNF‐α and IFN‐γ generally upregulate the expression and activation of MMPs in monocytes and macrophages.^78^ For example, in neutrophils, MMP‐9 is stored as gelatinase granules and it is released following stimulation with IL‐8 or TNF.^79^ TNF‐α is essential for MMP‐1 and MMP‐9 expression by monocyte in bronchial epithelial cell networks.^80^, ^81^, ^82^ IL‐12 is an important cytokine involved in both innate and adaptive immune responses.^83^ It is mainly secreted by macrophages, monocytes, dendritic and B cells in response to bacterial infection.^84^ It stimulates T‐ and NK cells to produce IFN‐γ and promotes the Th1 response.^85^ Recently, researchers have investigated the role of IL‐12 in secretion of various MMPs. Miao and colleagues described that IL‐12 significantly increased the mRNA and protein level expressions of MMP‐1, MMP‐3 and MMP‐13, while it downregulated MMP‐2 and MMP‐9 in the human periodontal ligament fibroblasts.^86^ This IL‐12‐mediated regulation of MMPs was NF‐κB‐dependent. However, IL‐12 treatment had no significant effect on the mRNA and protein levels of TIMP‐1 and TIMP‐2. The other possible mechanisms of IL‐12‐mediated transcriptional regulation of MMPs in this study have also been discussed recently.^87^

Historically, Th1 cells have been believed to be essential in the control of *Mtb* infection but now Th17 cells have been recognized as critical players in *Mtb* control.^88^ Th17 cells produce IL‐17, a pro‐inflammatory cytokine that functions to induce the secretion of diverse cytokines, chemokines, anti‐microbial peptides and MMPs.^89^ In macaques, sterile granulomas had a higher frequency of T cells producing IL‐17 and pulmonary delivery of BCG vaccine triggers a mucosal immune response orchestrated by IL‐17.^90^, ^91^ In a study in the China involving Han population, genetic polymorphisms in IL‐17A and IL‐17F were related to host susceptibility to TB and infection with hypervirulent W‐Beijing strain HN878 required IL‐17 for early immunity.^92^, ^93^ Mice with genetically incapacitated IL‐17 receptor are more susceptible to *Mtb*, despite a normal Th1 response.^94^ Similarly, IL‐17 knockout mice failed to develop mature granulomas after BCG infection and showed diminished protection from virulent *Mtb*.^95^ So IL‐17 has a well‐established role in host defence against TB, but its role in TB‐driven tissue damage was unknown. A recent study by Singh et al discovered the role of this cytokine in regulation of MMP secretion by using biopsies from patients having pulmonary TB, patient's bronchoalveolar lavage fluid (BALF) and primary human airway epithelial cells.^96^ IL‐17 was expressed in TB patient granulomas, and MMP‐3 was expressed in adjacent pulmonary epithelial cell, while IL‐17 exhibited a concentration‐dependent effect on MMP‐3 secretion. On the other hand, IL‐17 decreased the secretion of MMP‐9. Moreover, this IL‐17‐driven MMP‐3 upregulation was p38 MAP kinase‐dependent.

### 3.4 NF‐κB and MAPK regulation of MMPs

**Figure 2:**
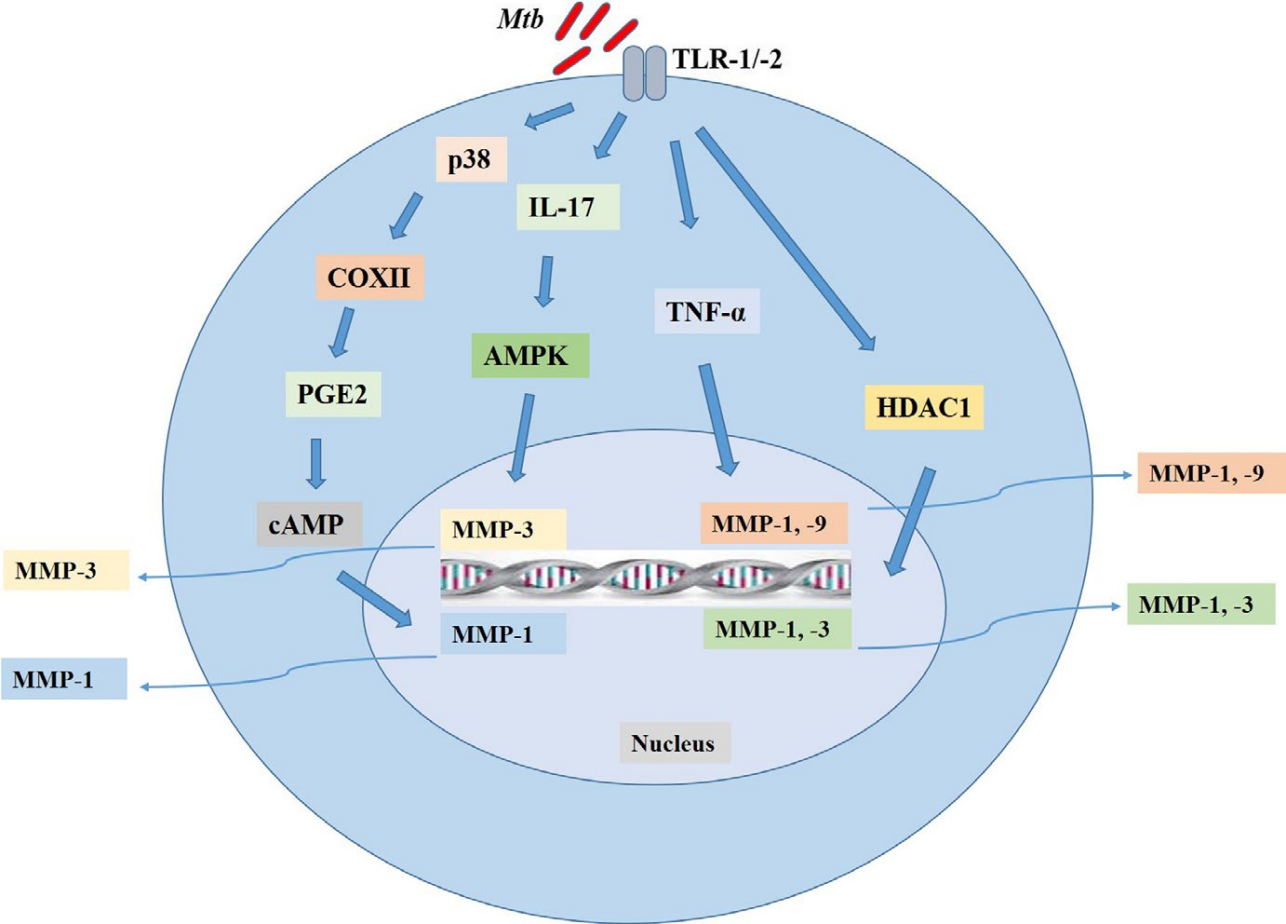
Expression and regulation of MMPs in TB. In *Mtb* infection, there is upregulation of many MMPs which are contributing to TB pathology. The expression and secretion of MMPs are strictly regulated as excess of these enzymes may cause tissue destruction. Many studies have investigated these signalling pathways. P38/COXII/PGE2/cAMP is well‐established signalling cascade involved in the regulation of MMP‐1. In a recent study, IL‐17‐mediated regulation of MMP‐3 was dependent on MAPK. In addition, TNF‐α and HDAC1 can also regulate MMP‐1 and MMP‐9, and MMP‐1 and MMP‐3, respectively. As the excess of these MMPs may be detrimental for host and may exaggerate the TB pathology, therefore, inhibition of these signalling pathways may provide a new avenue for host‐directed therapy in TB

In *Mtb* infection, multiple pathways are activated which together regulate MMP secretion (Figure 2). It has been reported that key transcriptional regulators of MMP expression in TB are NF‐κB and STAT3.^30^, ^97^, ^98^ TIMP‐1 lacks an NF‐κB promoter binding site, and so, NF‐kB signalling may regulate the MMP/TIMP expression.^55^ Recently, Miao and colleagues reported that IL‐12 significantly increases the mRNA and protein level expressions of MMP‐1, MMP‐3 and MMP‐13, and downregulates MMP‐2 and MMP‐9 in the human periodontal ligament fibroblasts.^86^ NF‐κB signalling was involved in this IL‐12‐mediated regulation of MMPs. Other studies have also reported the same findings.^99^ Intracellularly, MMP secretion is also regulated by the prostaglandin (PG) and mitogen‐activated protein kinase (MAPK) signal transduction pathways both in case of direct infection of *Mtb* or through intercellular networks.^66^, ^98^ p38 MAPK pathway activation has multiple downstream effects, its activation leads to COXII accumulation, prostaglandin (PG) E2 and cAMP activation and ultimately upregulates MMP‐1 secretion [66]. In addition, *Mtb* itself produces cAMP which can be utilized to undermine the host immune response.^100^ On the other hand, this pathogen‐derived cAMP may contribute to increased MMP secretion. In a recent study, p38 MAPK signalling was involved in IL‐17‐driven MMP‐3 upregulation in *Mtb* infection.^96^ AMPK also regulates neutrophil‐derived MMP‐8 secretion in TB.^101^ Besides these pathways, Moores et al investigated the role of histone acetylation changes in *Mtb*‐induced MMP secretion.^102^ Silencing of HDAC1 by using siRNA resulted in downregulation of MMP‐3 expression, but this silencing had no effect on MMP‐1, which shows epigenetic modification of histone acetylation also plays a role in expression of MMP‐3. Taken together, multiple pathways are involved in MMP regulation but it has yet to be established that which signalling pathway can be effectively targeted to minimize the TB pathology.

## 4 ROLE OF MMPS IN TB IMMUNOPATHOLOGY

### 4.1 Role of MMPs in pulmonary granuloma and cavitation

Tuberculosis is primarily a disease of lung, and following infection with *Mtb*, pulmonary granulomas are formed in humans and develop heterogeneous microenvironments, often featuring hypoxia and central necrosis.^103^ The same lesions can be recapitulated in non‐human primate and rabbit models of the disease.^104^ Historically, pulmonary granuloma formation has been considered as a host protective response; however, recent studies suggest that *Mtb* uses secreted virulence factors to induce granuloma formation to create a favourable niche for its dissemination and replication.^105^, ^106^ Moreover, recent studies have demonstrated a wide variation in the distribution of drugs within TB granulomas, with very few agents able to penetrate the central regions of the granuloma.^107^ This differential ability of drugs to penetrate TB granulomas has been incorporated into modern TB drug development programmes to select a more efficient combination.^108^ However, the mechanisms contributing to this differential penetration of drugs are not fully understood yet, and novel approaches to improve TB drug delivery and efficacy are urgently needed. The standard treatment of TB has remained unchanged for many decades, and multidrug‐ and extensively drug‐resistant strains are emerging progressively, leading to high mortality rates among patients even after commencement of TB treatment.^109^, ^110^, ^111^, ^112^ Therefore, it urges the development of new drugs to accomplish the sustainable development goals, aiming to reduce 90% of TB incidence rate by 2030.^113^

The role of MMPs in pulmonary physiology and pathology is gaining attention, and several studies show that they are associated with lung tissue destruction and inflammatory lung disorders including chronic obstructive pulmonary disease (COPD) and emphysema. *Mtb* infection induces the production of MMPs both in vitro and in vivo.^40^, ^114^ Many MMPs, specifically MMP‐1, have been shown to contribute in TB pathology in human lungs.^82^ Although the primary function of MMPs is thought to be matrix cleavage and tissue remodelling, many evidences suggest these enzymes also play a major role in angiogenesis, cell motility, apoptosis, regulating immunity, inflammation and host defences.^115^ Moreover, several MMPs through proteolytic activity can modulate the functions of cytokines and chemokines including IFN‐γ, IL‐1β, TNF‐α, CXCL8 and CCL7. Thus, MMPs, besides tissue destruction, can regulate chemokine gradients and leucocyte recruitments to the sites of inflammation.^115^

**Figure 3:**
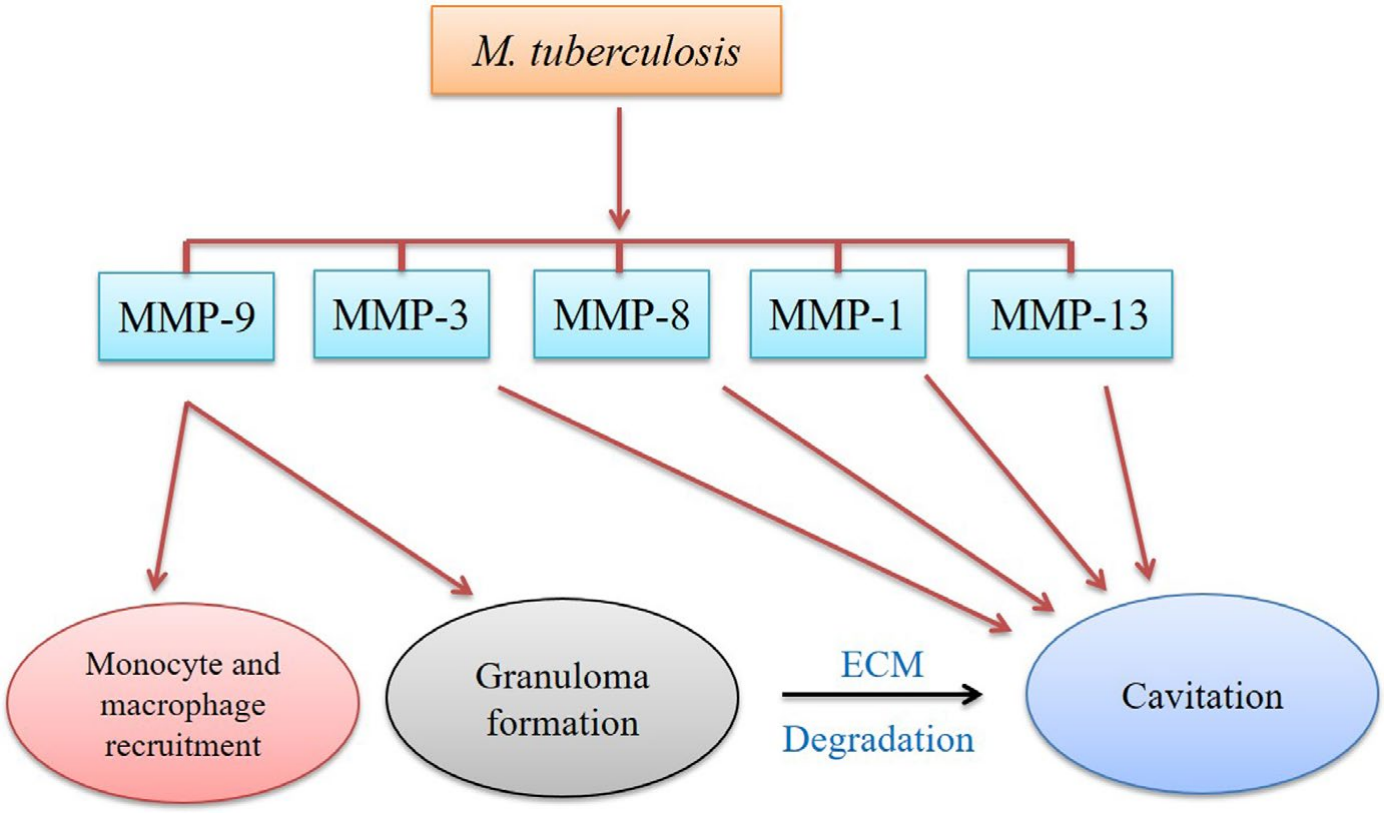
Role of MMPs in pulmonary granuloma and cavitation. Many studies have reported the increased expression of various MMPs in TB. Each of these MMPs has immunomodulatory role in the pathogenesis of TB. But MMP‐1, MMP‐3 and MMP‐9 are the most widely studied MMPs in TB, and their role in TB development has been well demonstrated. MMP‐9 upregulation is related to the recruitment of monocytes and macrophages at the site of infection, and these cells are main players of host innate immune response and are also crucial for granuloma formation. Upregulation of MMP‐9 in *Mtb* infection and as a result lung tissue remodelling is considered as mycobacterial strategy to create a suitable environment in host lung tissues. In contrast to MMP‐9, MMP‐1 degrades ECM as its primary function. ECM destruction leads to necrosis and cavitation thereby providing an immune‐privileged site for bacterial proliferation. MMPs such as MMP‐3, MMP‐8, MMP‐13 and others are also involved in tissue remodelling in TB. Detailed knowledge of these MMPs’ expression and function may help to devise a strategy to control the replication and spread of *Mtb* in host tissues

Many studies have validated the role of MMPs in pulmonary granuloma formation. Mice treated with BB‐94, a broad‐spectrum inhibitor of MMPs, revealed either a delay in granuloma induction or form smaller granulomas with more collagen content.^116^, ^117^ These studies, for the first time, suggested that MMPs regulate cell migration and granuloma formation after *Mtb* infection. Consistent with these findings by using various MMP inhibitors, MMP‐9‐deficient mice also showed a reduction in macrophage recruitment to the lungs and developed comparatively smaller granulomas.^21^ Similarly, Volkman and colleagues discovered the molecular mechanisms that *Mtb* uses to induce granuloma formation.^118^ They showed that the 6‐kDa early secreted antigenic target (ESAT‐6) induces MMP‐9 in epithelial cells adjacent to infected macrophages. This upregulation of MMP‐9 is related to the recruitment of monocytes and macrophages essential for granuloma formation. These studies provided the basis for the emerging paradigm that MMPs remodel lung tissue and initiate granuloma formation. Upregulation of MMPs in *Mtb* infection may be considered as mycobacterial strategy to multiplicate and spread in the host lung tissues. Along with granuloma, pulmonary tissue destruction is a hallmark of TB pathology leading to morbidity, mortality and transmission of infection. ECM destruction allows necrosis and cavitation, and thereby creates an immune‐privileged site, wherein the bacilli can proliferate and eventually spread to new hosts.^119^ In contrast to MMP‐9 which plays a role in ECM deposition and granuloma formation, MMP‐1 is believed to degrade ECM as its primary function, thus contributing in spread of *Mtb* and cavitary disease development (Figure 3).^22^ However, the pathogenesis and role of other MMPs are incompletely understood.^120^ The understanding of MMP role is important for designing the rational therapies to minimize immune‐mediated host damage and improve outcomes in TB.

### 4.2 Animal models of pulmonary granuloma and cavitation

Mouse, rabbit and guinea pig are most commonly used animal models of TB.^121^, ^122^ These species of animals have contributed a lot to the understanding of TB immunopathology. In recent years, majority of in vivo* Mtb* investigations have been carried out by using mouse model, and different mouse strains and different infection methods have been created.^123^ *Mtb* infection has been reported to upregulate MMP‐9 expression in *Mtb*‐infected mouse.^124^ In another study, MMP‐9 knockout mice exhibited reduced cellular recruitment to the lung granuloma.^125^ These studies implicate that MMP‐9 is required for recruitment of macrophages and tissue remodelling in *Mtb* infection of mice. But, unfortunately, mouse model of TB fails to develop the well‐characterized granuloma structure and cavitation which is commonly seen in human TB.^7^, ^126^ Moreover, mice do not express human MMP‐1 orthologue which, along with other MMPs, causes tissue destruction and transmission in disseminated human granuloma.^34^ As MMP‐1 is considered as dominant collagenase driving matrix destruction, therefore, the C57BL6 mouse model of TB has limited use to dissect the role of MMPs in *Mtb*‐driven immunopathology. However, C3HeB/FeJ mice have been shown to develop granuloma and occasional cavitation. 127, 128 These mouse granuloma studies lack the information regarding expression of MMPs and their role in granuloma formation. In future, a thorough understanding of MMP expression in *Mtb*‐infected C3HeB/FeJ mice could increase the utility of this animal model.

Other animal models such as guinea pig, rabbits and non‐human primates (NHPs) are preferred which exhibit caseation necrosis as seen in human.^129^, ^130^, ^131^ Guinea pigs have contributed significantly to understand the immunopathology of pulmonary TB. Although the guinea pigs are relatively economical model and produce TB lesions more similar to human in terms of lung pathology, even then they lack some characteristics features of human TB.^132^ In this regard, rabbits develop cavitary TB same as seen in human and offer a good opportunity to study the factors causing this type of disease. Moreover, rabbits are also susceptible to bovine TB caused by *M bovis*.^133^ Rabbit model is suitable for the investigation related to drug penetration and distribution in the lung tissue, and also to evaluate the response to chemotherapy.^134^ Adjunctive host‐directed therapies have evolved as a new approach to enhance the efficacy of conventional anti‐microbials against TB. In a study on rabbit model, expression of MMP‐1, MMP‐12 and MMP‐14 was significantly reduced in the lungs of CC‐11050 (a phosphodiesterase‐4 inhibitor)‐treated rabbits as compared to the *Mtb*‐infected untreated animals.^135^ In a previous study by the same research group, INH treatment of *Mtb*‐infected rabbits significantly reduced the expression of MMP genes including MMP‐14, MMP‐12, MMP‐2, MMP‐3, MMP‐9, MMP‐1 and MMP‐13 as compared to the untreated infected controls. The expression of MMP‐1, MMP‐3 and MMP‐12 was further significantly reduced in the animals receiving combined treatment of INH and CC‐3052, a phosphodiesterase‐4 inhibitor, compared with the group receiving INH treatment alone.^136^

Zebrafish model has been used to study MMP expression and granuloma in TB. The role of MMP‐9 has been reported in modulating cellular recruitment to the granuloma, and reduced expression of MMP‐9 resulted in smaller granulomas.^137^ In a related study, virulent *M marinum* significantly upregulated the expression of MMP‐9, MMP‐13 and MMP‐14 as compared to an attenuated strain.^138^ These studies suggest the key role of MMP activity in the pathogenesis of mycobacterial infection. Besides these TB models, non‐human primates (NHPs) such as the cynomolgus macaque have been used to replicate human TB lesions.^139^ NHPs have a close evolutionary relationship with humans and produce TB disease with clinical findings and lesions very similar to those of humans. Macaques develop granuloma types as seen in humans with the presence of classical caseous pulmonary granuloma. Moreover, macaques also exhibit other types of lung lesions such as non‐necrotizing granulomas, calcification, cavitation, consolidations and interstitial fibrosis.^140^ These lesions have been reported recently in *Mtb*‐infected cynomolgus macaques of Chinese origin.^141^ In a previous study involving microarray analysis, multiple MMPs were upregulated in *Mtb*‐infected macaques. MMP‐1 was the most highly expressed, while MMP‐2, MMP‐7, MMP‐9, MMP‐14 and MMP‐25 expression was also induced by *Mtb* infection.^142^ Luckily, antigens of cynomolgus macaque give cross‐reactivity with immunologic reagents made for human cells and tissue, thus making the immunohistochemical investigation easier in these TB models. The main disadvantages of this TB model are high cost and more space requirement in BSL3 facilities.

### 4.3 Role of MMPs in TB/HIV co‐infection

It is believed that almost half of patients surviving pulmonary TB with apparent recovery suffer substantial pulmonary impairment.^143^, ^144^ Furthermore, cured pulmonary TB is a major cause of chronic lung disease globally.^145^ In 2017, TB caused about 1.3 million deaths among HIV‐negative people and there were additional 0.30 million deaths from TB among HIV‐positive people.^146^ Among HIV‐infected patients, the mechanisms leading to prolonged pulmonary morbidity after successful TB treatment are not well understood.^143^, ^144^ Previous reports show that the relative risk of active TB doubles during the first year of HIV infection even when the CD4 counts are still conserved. This risk of active TB continues to increase in the upcoming years as the CD4 number is decreased.^147^ These findings are also in line with the central role of cellular immune response in structural lung damage and cavity formation in TB.^148^ Mechanistically, MMPs are the main players in TB‐associated lung tissue destruction.^18^, ^34^ However, lower MMP levels and reduced TB‐associated lung damage were seen radiographically in TB/HIV co‐infection.^64^ Variable MMP activity has been shown in HIV‐1‐infected and HIV‐1‐uninfected TB individuals. HIV‐1 infection in TB patients leads to decreased pulmonary MMP concentrations and reduced cavitary lesions.^63^, ^149^

These findings suggest that patients having TB/HIV co‐infection may have less lung matrix destruction than those with TB infection alone, but other studies have reported conflicting results.^145^ Antiretroviral therapy (ART) is a critical part of HIV/TB co‐infection treatment. Early start of ART in patients having CD4^+^ T cell less than 50 per cubic millimetre increased the chances of AIDS‐free survival, while in another study the treatment was associated with pulmonary airway obstruction.^150^, ^151^ Immune response restoration in HIV may cause lung damage and consequently lead to immune reconstitution inflammatory syndrome (IRIS). IRIS usually occurs during the initial months of ART and is commonly associated with TB. This TB‐IRIS is associated with a distinct pattern of MMP gene expression and secretion. In a study, HIV/TB co‐infected adults, and in stimulated cultures, secretion of MMP‐1, MMP‐3, MMP‐7 and MMP‐10 was higher in TB‐IRIS than in controls. Corticosteroid therapy for two weeks resulted in non‐significant reduction in MMP‐7 in serum, while the secretion of other MMPs was not affected.^152^ While, in another study, ART induced the increased expression of MMP‐8,^153^ this increased expression of MMP‐8 might cause lung damage.

Studies have shown that ART causes virologic suppression, but, on the hand, it may lead to early immunologic failure which is associated with early mortality after ART initiation in advanced HIV/tuberculosis.^154^, ^155^ Therefore, interventions to decrease inflammation and promote cellular immune recovery during ART may be helpful in patients co‐infected with HIV/TB. A possible limitation of the studies addressing HIV‐mediated lung damage in TB is that the effects of TB treatment on cellular immune response have not been evaluated in details. Further studies would elaborate the association between lung damage and TB‐IRIS and also the mechanisms involved; whereby, immune response restoration impairs pulmonary function.

## 5 INHIBITION OF MMPS: UNLEASHING THEIR THERAPEUTIC ROLES

Host‐directed therapy with MMP inhibitors has been investigated in several inflammatory conditions such as multiple sclerosis.^156^ Encouraging results have been obtained in experimental models of meningococcal and pneumococcal meningitis, where MMP inhibition resulted in decreased morbidity and mortality.^157^, ^158^ Similarly, Oehlers et al showed that vascular endothelial growth factor (VEGF) inhibitors in combination with rifampin reduce *M* *marinum* burden in zebrafish.^159^

In TB, host‐directed therapies are evolving as a novel therapeutic paradigm and many research groups have used various MMP inhibitors to study the immunopathology of TB.^160^, ^161^ Doxycycline is an anti‐mycobacterial antibiotic, and it is the only FDA‐approved MMP inhibitor. It has also shown promising effects on TB treatment by inhibiting the mycobacterial growth in animal and in vitro models of the disease.^64^ In a previous study, doxycycline suppressed TB‐dependent MMP‐1 and MMP‐9 secretions from primary human macrophages and epithelial cells. Moreover, doxycycline treatment decreased MMP activity in a cellular model and suppressed mycobacterial growth in vitro and in guinea pigs.^63^ Neutrophil‐derived MMP‐8 may also drive lung cavitation, morbidity and death.^101^ Therefore, MMP‐8 inhibition may be a potential target to abolish excessive host tissue destruction as MMP‐8 inhibition in a murine model of lung injury improved the outcomes of the therapy.^162^

Marimastat (BB‐2516) is another specific MMP inhibitor, a collagen‐peptidomimetic drug that targets the active site zinc atom of several MMPs, thereby preventing their activity. Marimastat is well tolerated in in vivo and has been tested to prevent cancer metastasis.^163^ Though, the anti‐neoplastic properties of the drug are well known in clinical trials, however, it has not yet been approved for clinical use due to its side effects on musculoskeletal system.^18^ Recently, some research groups have used this and other related drugs to inhibit MMP expression in TB. Parasa and colleagues used lung tissue model of TB comprising of human lung‐derived cells and primary human monocyte‐derived macrophages.^12^ Inhibition of MMPs by marimastat reduced both granuloma formation and bacterial load in *Mtb* infection, suggesting that MMP‐targeting intervention could be considered as a supportive therapy in TB treatment. Administration of marimastat alone did not show protective response in *Mtb‐*infected C57BL/6J mice; however, when administered in combination with either rifampin or isoniazid as adjunctive treatment, it increased the drug exposure in infected lung tissues and caused a reduction in bacterial burden of lungs when compared with animals treated with rifampin or isoniazid alone.^11^ In contrast, administration of adjunctive cipemastat, an orally available potent inhibitor of MMP‐7, increased the frequency of cavitation, immunopathology and mortality in *Mtb‐*infected C3HeB/FeJ.^164^ The same research group evaluated the use of anti‐MMP‐9 antibody in combination with first‐line drugs of TB treatment and reported significantly reduced relapse rates of TB in C3HeB/FeJ mice as compared with the mice receiving standard therapy alone.^165^ Consequently, MMP inhibition has divergent effects when administered alone or in combination with first‐line TB treatment. The findings highlight the importance of exploiting strategies that improve the efficacy of existing drugs by increasing the effectiveness of the anti‐TB therapy.

## 6 CONCLUSIONS AND FUTURE PERSPECTIVES

Taken together, a promising model for the role of MMPs in TB is that *Mtb* induces lung tissue remodelling and granuloma formation through upregulation of MMPs. Intact granuloma is thought to be beneficial to the host as it keeps the pathogen under check and prevents its spread. Reactivation of the infection and increased secretion of MMP‐1 result in pulmonary matrix degradation and cavitation. However, regulation and role of specific MMPs during various stages of *Mtb* infection remain to be explored. Nonetheless, role of kallikrein‐kinin system in MMP regulation in *Mtb* infection remains completely unknown. Recently, most of the publications, using various animal models of TB, suggest MMPs as viable therapeutic targets. Adjunctive treatment with MMP inhibitors along with front‐line TB drugs including isoniazid and rifampin significantly reduces *Mtb* survival in the lungs by preventing maturation of granulomas and also minimizes the matrix degradation and cavitary lesions. Current TB therapeutic regimens need multiple drugs and have to be taken for long times; therefore, they impose other challenges such as non‐compliance and emergence of the drug‐resistant *Mtb* strains. Given these challenges, MMP targeting may provide a reliable approach to increase the potency of current anti‐TB drugs.

## CONFLICT OF INTEREST

All authors declare no conflict of interest.

## AUTHORS' CONTRIBUTIONS

NS collected the data and wrote the manuscript. TH and MHM helped for figure and table compilation. XZ gave the idea behind the manuscript compilation. DZ reviewed the article before final submission. All authors read and approved the final manuscript.
